# Locally advanced high-risk HPV related oropharyngeal squamous cell carcinoma (OPSCC); have we forgotten it is a different disease?

**DOI:** 10.1186/s41199-018-0035-7

**Published:** 2018-10-03

**Authors:** Nabil F Saba, Shuli Li, Zain A Hussain, Rathan Subramanian, Joseph A Califano, Christine H Chung

**Affiliations:** 1 Winship Cancer Institute, Department of Hematology and Medical Oncology, Winship Cancer Institute of Emory University, 1365Clifton Road, building C, Atlanta, GA 30322 USA; 2 Harvard TH Chan School of Public Health, Boston, USA; 3 Radiation Oncology, Yale Cancer Center, Yale School of Medicine, New Haven, USA; 4 Department of Radiology, UT Southwestern University, Dallas, USA; 5 Division of Otolaryngology and Head and Neck Surgery, UC San Diego, San Diego, USA; 6 Head and Neck and Endocrine Oncology, Moffitt Cancer Center, Tampa, USA

## Abstract

HPV related OPSCC has a distinct behavior and improved outcome. As immunotherapy has recently evolved into a new standard for advanced (SCCHN), trials for high-risk SCCHN have trended to encompass both HPV related and unrelated diseases. In this invited editorial, we question the wisdom of this approach and argue for the design of trials focused specifically on HPV related locally advanced oropharyngeal squamous cell carcinoma as a unique disease entity.

## Editorial

Since the MACH meta-analysis and early phase III trials, the treatment of locally advanced SCCHN have relied on chemoradiotherapy [1]. For more than a decade, we have known that the subset of HPV related OPSCC has a distinct behavior and improved outcome [2–4]. Initial de-intensification trials for this group included patients with different risk categories, such as Eastern Cooperative Group Trial 1308 (E1308) and the Radiation Therapy Oncology Group trial 1016 (RTOG 1016). Data from E1308, large retrospective series, and more recently AJCC 8th Edition have clearly revealed that patients with T4, N3 or > 10pk year smoking history carry a worse outcome [5, 6]. More recent de-intensification efforts have tended to exclude these patients.

As immunotherapy has recently evolved into a new standard for advanced HPV related and unrelated squamous cell carcinoma of the head and neck (SCCHN) [7], trials for high-risk SCCHN have also trended to encompass both diseases. These designs have relied mostly on adding an immune check point inhibitor (ICPI) to concurrent therapy, and/or, the use of ICPI in a maintenance approach. Possible influencing factors behind these trends include and may not be limited to, the apparent comparability in prognosis of the two diseases observed in older studies such as RTOG 0129 and 0522, in addition to the ardent desire in reaching rapid registration of novel agents in the competitive immunotherapy realm.

Even though the rationale for these approaches we believe is valid, the wisdom of encompassing both HPV related and unrelated disease is questionable. Assuming a similar outcome for diseases with different etiologies, presentations, pathologic and radiologic characteristics, degree of co-morbidities, clinical staging, and pattern of distant metastases we believe deserves a closer examination [8, 9]. Noteworthy here, is that the prognosis for the high-risk HPV related OPSCC is difficult to tease from RTOG 0129 and 0522, as these reports did not clearly dissect out this group and their designs preceded the HPV era. The mere fact that a similar reported outcome existed in these reports should therefore not justify assuming equivalence in prognosis and appropriate therapeutic approaches for two distinct diseases.

ECOG ACRIN EA3161 is a recently activated phase II/III trial examining the question of whether a maintenance approach with a single agent ICPI following definitive therapy would alter the progression-free survival (PFS) or overall survival (OS) for high-risk HPV related disease. As this study focuses on HPV related disease, it also avoids intensification of concurrent therapy and relies instead on abrogating the risk of distant metastases through a maintenance approach. We believe therefore EA3161 it uniquely positioned to answer an important question for a group of healthier patients with better prognosis yet equivalent risk of distant metastases.

It is likely that adding ICPI to the backbone standard concurrent regimens will be proven effective for locally advanced SCCHN soon. The questions of whether intensification of concurrent therapy for the HPV related group is the correct approach and the degree to which this group will benefit from a maintenance ICPI, we believe will continue to linger for some time to come. EA3161 is poised to answer these questions.

## Abbreviations

ACRIN: American College of Radiology Imaging Network
ECOG: Eastern Cooperative Oncology Group
